# Filling Teeth

**Published:** 1848-07

**Authors:** E. Taylor


### FILLING TEETH.

BY DR. E. TAYLOR.

*(Continued from No. 2.)*

Having the cavity and foil prepared as before stated, the patient well seated, the head thrown back and the chin up so as to afford a good light, and cautioned to hold it so until the filling is secured, we take a napkin and wipe the teeth on that range dry, then insert the corner of the napkin between the corner of the lip and the gums, carry it back past the salivary duct and coming around below cover the lower lip; this being held secure with one hand, preserves the dryness of the parts and the foil. If the cavity be large or irregular it is best to have a small syringe, (which should be made of silver or gold, and show its design,) and some tepid water with which to wash the cavity out, or a little pledget of cotton well saturated will answer the purpose very well, after which the cavity is to be well and thoroughly dried with pledgets of cotton or scraps of cotton or linnen cloth. The instrument I generally use for filling these cavities is a little curved, rather slim toward the end, squared with the edges rounded and wedge pointed. I usually have a little loose lump of foil rolled up with the end of my strip, suited to the size of the cavity, so as to be retained, stuck onto the end of my plugger and begin at the side of the cavity having the greatest inequality, if any such exist, but if not, I fill against the back part, folding the foil so as when carried to the bottom of the cavity the other end of the fold will be considerably outside, then doubling in and forcing it against the back as long as I can with much force work the point of my instrument in, I then take a small point and examine every part to be sure that it is compact throughout and always like to have enough foil firmly connected to form a full head that may be filed off. After it is well compacted take a point that will about cover the cavity that is cross-furrowed and compress it as long as it will yield, then file away the surplus foil; for this purpose, if a proper file be not to be had otherwise, the temper may be drawn from a round or half round file, and it bent to a proper shape and the temper restored. The edges of the plug will frequently need triming with a sharp pointed instrument and the filling should always be cut so that the antagonist tooth will not strike it first in closing the teeth, I usually then finish with a burnisher and like to have it so smooth that it looks like a piece of polished plate. Some, with more fancy, rub them up with Arkansite or Scotch stone, polishing powder, &c. This operation as I formerly stated, is one of the most simple we meet with in filling, yet comparatively few perform it perfectly so as to entirely preserve the tooth, but fail in some one or other of the important particulars.

The difficulties to be encountered in preparing and filling the side cavities are much greater, and such as to require much skill and ingenuity and judgment, and we may add patience and perseverance.

On the sides of the incisors we usually find the cavities difficult of access, and illy shaped. To overcome these difficulties the Dentist must set himself to work to prepare room to operate. Sometimes, particularly in young persons, where the edges are not broken, they may be wedged apart either with wooden wedges increased daily in thickness, or with strips of caoutchouc drawn between.– The former I regard as the better, as the latter is rather too violent and produces too much inflamation in the socket. We usually prefer the file for this purpose, especially if the decay has progressed to any considerable distance and left the edges of the tooth ragged or weak. Good judgment must be observed in preserving the shape of the tooth as well as possible and not have the space unseemingly or unnecessarily large, and more may be filed from the inner side where it will not show. Sometimes it is difficult to determine whether to remove the caries with the file or excavate and fill it.—If, however, it has penetrated much through the enamel, we generally prefer to fill it, as the shape and comfort of the tooth is best preserved in this manner. I think the fault most common with young operators is the fear of filing too much, which fear is too much fostered by the general prejudice and antipathy to the use of the file. It must, however, be regarded as a disagreeable necessity and submission required. The Dentist should have a well cultivated judgment of his own and firmness enough to carry out its dictates without wavering; as a hesitating course will loose him the confidence of his patient. We do not recommend making spaces large enough for conveniently working with large, awkward instruments, but the instruments should be finely tempered and delicately pointed, and large enough to have strength for the purpose designed.

In connection with the spoon-shaped cavities so frequently met with in these teeth, we also usually meet with a very considerable amount of sensibility. It has been an object in the profession to obtain some harmless specific to relieve this sensibility and Arsenic acid was for a time highly extolled for its virtues in this respect, but although it does in a few hours destroy the sensibility, yet its consequences in destroying the vitality of the tooth has caused its use to be almost if not entirely abandoned. Some claim much success from Creosote and some of the strong astringents, as Tannin, Nutgalls, Alum, &c. These may answer well sometimes, but I have not met with that success in their use as to induce me to continue them. The most successful treatment, I think, is a keen, sharp excavator carried to the bottom of the caries at once, and laid under the diseased part. Kindness and firmness must be observed to the patient as a little sympathy will often much strengthen the nervous system.

The shapes and positions of the cavities are so varied that it is impossible to describe the shape of the instruments necessary.—Those steel handled are now so cheap that the Dentist is inexcusable for not having a good supply, and a man is not fit to operate who has not ingenuity enough to adapt points to the necessities of his case. Suffice it to say that the caries must be thoroughly removed and the cavity properly shaped and the gold fully consolidated.—In these as in all other cases, we always leave the foil very prominent, so as after compressing, to leave enough to file the head of the plug smooth. The edges of the tooth should also be rounded off and the whole smoothed and polished up finely. A thin Arkansite, or some finely pulverized pumice stone on tape will serve well to rub up the surface of the filling.

Cavities in the sides of the Bicuspidati and Molares present about as much difficulty in shape, and more in position, being farther back in the mouth and more difficult to separate. The file is useful here also, although a cutting instrument, chisel shaped, or such as spoken of in connection with preparing the cavities in the centre of the Molares, may be used with much advantage, and is usually more expeditious and not so disagreeable to the patient. Taking a small portion of the enamel at a time it may be very readily cut away with the instruments, but I prefer a file to bring down the edges smooth. I usually leave the space narrower near the gum, so shaped as food, &c., is less apt to be retained, and the cavity more accessible to the plugger. The necessities of the case will better direct to the shape of the instruments than any description that I might be able to make. Some good strong points with furrowed sides or edges are very important in compressing the foil —compresses better and is not so liable to slip off. Variously shaped and situated cavities will present themselves around the necks of the teeth that will require all the skill that the most experienced are capable of, to encounter successfully, and in filling the lower teeth the saliva is very much in the way as well as the tongue. Dr. ——— has invented an instrument for holding down the tongue and surpressing the flow of saliva from the sublingual ducts, which is highly spoken of by those who have used it. I yet prefer a properly constructed speculum that the patient may hold with one hand. It consists of two parallel bars or wires, (silver,) about as thick as a goose quill, an inch and a half to two inches long and about an inch apart, connected at each end by a semi-circle, so as to pass over back of the dens sapientiæ and over the bicuspidati, the handle coming out over and down the chin some six or eight inches, so that the hand holding it will be out of the way of the operator.

Place under the tongue, over the sublingual ducts, a few folds of linen or cotton cloth, and also under each bar, passing on either side of the teeth pretty thick folds to absorb the saliva, and the patient may thus very conveniently hold the tongue and saliva at bay while the filling is being put in. I had intended saying something about filling teeth after the pulp has been destroyed, but it is so uncertain and ugly a piece of business that the less a Dentist has to do with it the better, as well for his own reputation as the comfort or real service to his patient.

